# Effects of a lifestyle modification programme to reduce the number of risk factors for metabolic syndrome: a randomised controlled trial

**DOI:** 10.1017/S1368980016001920

**Published:** 2016-07-29

**Authors:** Mariko Watanabe, Masako Yokotsuka, Kazue Yamaoka, Misa Adachi, Asuka Nemoto, Toshiro Tango

**Affiliations:** 1 Showa Women’s University, Tokyo, Japan; 2 Teikyo University Graduate School of Public Health, 2-11-1 Kaga, Itabashi-ku, Tokyo 173-8605, Japan; 3 Nutrition Support Network LLC, Kanagawa, Japan; 4 Center for Medical Statistics, Tokyo, Japan

**Keywords:** Metabolic syndrome, Risk, Reduction, Lifestyle education, Randomised controlled trial

## Abstract

**Objective:**

To determine the effectiveness of a personal support lifestyle education programme (PSMetS) for reducing risk factors in individuals with metabolic syndrome (MetS).

**Design:**

A two-arm randomised controlled trial.

**Setting:**

Companies in metropolitan Tokyo, Japan.

**Subjects:**

Male workers with diagnosed MetS or a high risk for MetS according to the Counselling Guidance Program, Japan (*n* 193).

**Results:**

The reduction in the number of risk factors for MetS (as defined according to the criteria published by the Japanese Ministry of Health, Labor and Welfare in April 2007 (MHLW-MetS)) in the PSMetS group was not significantly different from that in the usual care group by van Elteren’s test (baseline-adjusted *P*=0·075) for intention-to-treat (ITT), while it was significant (baseline-adjusted *P*=0·038) for per-protocol set (PPS). The proportion of MHLW-MetS was significantly different between groups by van Elteren’s test (baseline-adjusted *P*=0·031). Two components of MHLW-MetS showed significant reductions in the PSMetS group: waist circumference (baseline-adjusted *P*=0·001) and BMI (baseline-adjusted *P*=0·002). PPS and ITT analyses showed similar results.

**Conclusions:**

For male workers with MHLW-MetS or a high risk of MHLW-MetS, PSMetS reduced the number of risk factors for MHLW-MetS.

Metabolic syndrome (MetS) is a worldwide epidemic due to changes in diet and lifestyle^(^ ^1^ ^)^. The prevalence of MetS in Japan is 28·8 % in adult men and 10·4 % in adult women^(^ ^2^ ^)^. MetS is characterised by central obesity, dyslipidaemia, hyperglycaemia and hypertension, and substantially increases the risk of developing type 2 diabetes^(^ ^3^ ^)^, CVD^(^ ^4^ ^)^ and certain cancers^(^ ^5^ ^)^.

Although the definitions used for MetS vary slightly among ethnic groups^(^ ^6^ ^–^ ^9^ ^)^, the basic characteristics are the same. The Examination Committee of Criteria for ‘Metabolic Syndrome’ in Japan^(^ ^9^ ^)^ has undertaken research to focus therapeutic strategies on reducing the long-term risk of CVD. By considering the defining characteristics of MetS and their scope for practical usage in health promotion, a set of practical criteria (not for diagnosis but as a standard to ensure conformity) for MetS was proposed in the ‘Standard Health Check-up and Counselling Guidance Program’ by the Ministry of Health, Labor and Welfare in April 2007^(^ ^10^ ^)^. Hereafter, we refer to this definition of MetS as ‘MHLW-MetS’. In spite of these efforts, however, the prevalence of MetS continues to increase and the development of an effective lifestyle modification programme for Japanese people to combat MetS is urgently needed. A meta-analysis of randomised controlled trials (RCT) showed that lifestyle modification was effective in resolving MetS and reducing the severity of component abnormalities^(^ ^11^ ^)^. However, there have been few reports of the effectiveness of lifestyle modification programmes in European and Asian populations^(^ ^12^ ^–^ ^15^ ^)^, including Japanese participants^(^ ^16^ ^–^ ^19^ ^)^. Since an appropriate assessment of habitual dietary intake at each meal is important for the treatment of MetS, we developed the FFQW82^(^ ^20^ ^,^ ^21^ ^)^ for the purpose of providing individuals with an awareness of their dietary problems. We developed a personal support and lifestyle education programme (PSMetS) for the treatment of MetS and reported the effectiveness of the programme for preventing^(^ ^22^ ^)^ and improving type 2 diabetes mellitus^(^ ^23^ ^,^ ^24^ ^)^. The aim of the present study was to assess the effectiveness of PSMetS in reducing the number of risk factors for MHLW-MetS compared with the usual care group at 1 year after baseline, in Japanese male workers, by an RCT.

The study was conducted according to the guidelines laid down in the Declaration of Helsinki and all procedures involving human subjects/patients were approved by the Medical Ethics Committee of the Showa Women’s University in 2008 (No. 08-02).

## Methods

### Study design

The study was a two-arm, parallel-group RCT (trial registration code: UMIN 000008560).

### Participants

Male workers aged 25–64 years with MHLW-MetS or high risk for MHLW-MetS described in the outcome session were recruited from workers in Tokyo, Japan. The participating companies (the company sizes varied from small to medium) were recruited through the Chamber of Commerce and Industry and networks of exchange meetings among company managers. The company managers asked the occupational doctor or nurse working at each company to participate in the survey. The study participants were office workers, products sales workers, automatic line workers, shift workers and marketing workers. They had participated in a standard health check-up between June 2010 and December 2013 and were categorised according to the practical criteria for MHLW-MetS in Japan^(^ ^10^ ^)^.

The definition of MHLW-MetS includes the following five risk factors.

**R1.**Central obesity: waist circumference ≥85 cm and/or BMI ≥25·0 kg/m^2^.

**R2.** Hyperglycaemia: fasting plasma glucose ≥100 mg/dl and/or glycated Hb ≥5·6 % (National Glycohemoglobin Standardization Prorgam).

**R3.** Dyslipidaemia: TAG ≥150 mg/dl and/or HDL cholesterol <40 mg/dl.

**R4.** Hypertension: systolic blood pressure ≥130 mmHg and/or diastolic blood pressure ≥85 mmHg.

**R5.** Smoking habit: currently smoking (assessed only for individuals having one or more of the risk factors R2 to R4).

(Note: although R5 is used as a conditional risk factor, we treated it as an independent risk factor in this classification, since smoking cessation is very important for preventing non-communicable diseases and improving health.)

Using R1 to R5, the MHLW-MetS level, such as ‘specific health check-up’ (MHLW-MetS) or ‘specific health guidance’ (high risk for MHLW-MetS), was defined for each individual as shown in Table 1. The key differences among the MHLW-MetS, the National Cholesterol Education Program Adult Treatment Panel III MetS definition (ATPIII-MetS) and the Japanese MetS definition (J-MetS) were as follows. Central obesity was defined differently among the MetS definitions. J-MetS and MHLW-MetS introduced abdominal obesity as a prerequisite of the diagnosis of MetS, with particular emphasis on waist measurement (MHLW-MetS also used BMI). In contrast, ATPIII-MetS treated abdominal obesity as one of the risk factors of MetS. Further, J-MetS and ATPIII-MetS included serum TAG and HDL cholesterol levels as independent risk factors, but MHLW-MetS treated these metabolic parameters as a single risk factor (dyslipidaemia).

**Table 1:** Classification criteria of MHLW-MetS level (MHLW-MetS or high risk of MHLW-MetS)

Central obesity (R1)	Number of other risk factors (R2, R3, R4)	Smoking (R5)	MHLW-MetS (‘specific health check-up’) or high risk of MHLW-MetS (‘specific health guidance’)
Based on waist circumference	≥2	–	MHLW-MetS
	1	Yes	MHLW-MetS
		No	High risk of MHLW-MetS
Based on BMI	3	–	MHLW-MetS
	2	Yes	MHLW-MetS
		No	High risk of MHLW-MetS
	1	–	High risk of MHLW-MetS

MHLW-MetS, the Ministry of Health, Labor and Welfare definition of metabolic syndrome^(^ ^10^ ^)^; R1–R5, risk factors.

Participants who had started medication for CVD, diabetes mellitus, hypertension or dyslipidaemia within the previous 3 months, who refused to give informed consent or to respond to the FFQW82 or the lifestyle and behaviour check sheet, and who had other chronic illnesses (CVD, diabetes, hypertension, dyslipidaemia and renal failure) were excluded. These participants were recruited during their annual specific health assessments and received the intervention for 9 months (6 months of intensive care and 3 months of less intensive care) and follow-up for 3 months (12 months in total). All participants received the details of the trial and were asked to give written consent.

### Randomisation, allocation concealment and blinding

Eligible participants were randomly assigned using a permuted-block technique with the use of a randomisation list (random permutated blocks with block size of 4^(^ ^25^ ^)^) with stratification by MHLW-MetS level (MHLW-MetS or high risk for MHLW-MetS) into the PSMetS and usual care groups. The study biostatistician (K.Y.) generated the randomisation list and the person in charge of allocation (M.Y.) of the PSMetS project (at Showa Women’s University, Japan) allocated the participants to groups according to the randomisation list. The randomisation took place after the baseline measures were taken. Whether the participants fulfilled the criteria for MHLW-MetS or not was double-checked by the project leader (M.W.).

Due to the nature of the treatment, it was not possible to blind participants to lifestyle education. However, team members responsible for data management, with the exception of the project coordinator, remained blind to treatment assignment until all the data were recorded after the follow-up was completed. To minimise the risk of bias, strict protocols for follow-up assessment procedures including data management were developed and research assistants were trained to adhere to these protocols.

### Intervention (PSMetS programme)

The PSMetS programme was based on strategies described in previous studies^(^ ^22^ ^–^ ^24^ ^)^ and a counselling guidance programme^(^ ^10^ ^)^. The PSMetS programme was composed of two individual face-to-face counselling sessions (30 min each) and six telephone calls (5–10 min each) by a registered dietitian over the 9-month intervention period, which included 6 months of intensive care. At the first face-to-face counselling session, the registered dietitian asked the participant to set a goal for reducing his number of clinical risk factors for MHLW-MetS after 12 months. The registered dietitian provided advice based on the information obtained by his/her assessments of the FFQW82 and the lifestyle and behaviour check sheet (both assessments were performed before randomisation). The lifestyle and behaviour check sheet was composed of five questions on the topics of: (i) participants’ consciousness of their own health status; (ii) the amount of physical activity they undertake; (iii) their eating behaviour for reducing excessive energy intakes at dinner; (iv) the proportion of meals at which they consume two portions of vegetables; and (v) the frequency at which they eat staple foods per week. The participants in the PSMetS group were asked to respond to the dietary assessment sheet in addition to these questions at the first face-to-face counselling session. The participants also set one or two goals to improve their diets, based on the dietary assessment sheet and according to the advice provided by the registered dietitian. During the telephone counselling sessions, the registered dietitian discussed with the participants their progress towards attaining their goals. When the participant had attained his initial goal(s), the registered dietitian asked the participant to set a new goal. When the participant had not attained his goal(s), the registered dietitian gave further advice when necessary. The step-by-step counselling was individualised.

Through these processes, each participant received specific education on topics such as how to reduce energy intake at dinner, reduce the intake of high-fat foods, increase the intake of vegetables and perform daily physical activities. Education on smoking cessation was also included in the counselling sessions by notifying the participants that smoking cessation is very important for preventing non-communicable diseases and improving health.

### Usual care

Participants in the usual care group received general advice for the control of MetS by a doctor at the health check-up centre, as well as a report of their dietary assessment made using the FFQW82 by a registered dietitian working with the companies where participants were recruited.

### Training of health professionals

The participating dietitians received training to gain experience of the intervention protocol under supervision by the project team.

### Outcomes

The primary outcome was the number of risk factors for MHLW-MetS including the five risk factors (R1–R5) described in the ‘Participants’ section above.

The alternative definitions for MetS, ATPIII-MetS (2001)^(^ ^8^ ^)^ and J-MetS (2005)^(^ ^9^ ^)^, were also examined as secondary outcomes. Roughly speaking, the MHLW-MetS corresponds to ATPIII-MetS or J-MetS and a high risk of MHLW-MetS corresponds to ‘high risk for MetS’. MHLW-MetS and J-MetS assume ‘central obesity’ as a necessary condition, but ATPIII-MetS does not. The other secondary outcomes were the proportion of participants with MHLW-MetS, the values of each component of MHLW-MetS (waist circumference, BMI, TAG, HDL cholesterol, systolic blood pressure, diastolic blood pressure, glucose intolerance and current smoking), and dietary intakes of energy and nutrients as calculated using the FFQW82 (Table 2). The five items of the lifestyle and behaviour check sheet were also examined as secondary outcomes (see Table 5). Health consciousness was asked with five response categories (‘always’ to ‘not at all’). The other four questions were each asked with six response categories (‘did not at all or do not want to do’, ‘cannot yet but want to do’, ‘1–2 times per week’, ‘3 times per week’, ‘4–5 times per week but continuing less than 6 months’ and ‘4–5 times per week and continuing for 6 months’). The item of ‘physical activity undertaken per week’ was assessed by asking about the ‘intention to move the body routinely, such as to use the stairs or walk’. ‘Frequency of eating staple foods’ was asked by displaying photographs of rice served in a bowl. ‘Frequency of two portions of vegetables intake at each meal’ was assessed by displaying a quantity (a small bowl) of vegetables and asking whether that quantity was consumed two or more times out of every three meals.

**Table 2:** Baseline characteristics of the participants with MHLW-MetS allocated to the intervention (PSMetS) group or the control group (*n* 193); male workers from nine companies in metropolitan Tokyo, Japan, enrolled June 2010–December 2013

	PSMetS group (*n* 96)		Control group (*n* 97)	
Variable	Mean, median or *n*	sd, IQR or %	Mean, median or *n*	sd, IQR or %
Age (years), mean and sd	40·7	9·5	41·4	8·5
No. of risk factors for MHLW-MetS, mean and sd	4·3	0·9	4·1	0·9
Components of MetS				
Waist circumference (cm), mean and sd	94·6	8·7	92·7	6·8
BMI (kg/m^2^), mean and sd	27·9	3·6	27·1	3·4
TAG (mg/dl), median and IQR	150·0	96–218	126·0	92–189
HDL cholesterol (mg/dl), mean and sd	50·7	10·8	51·9	11·5
Systolic blood pressure (mmHg), mean and sd	128·1	13·5	126·4	16·9
Diastolic blood pressure (mmHg), mean and sd	81·4	10·8	79·9	13
Glucose intolerance*, *n* and %	56	58	57	59
Smoking status (current), *n* and %	30	31	40	41
MHLW-MetS, *n* and %	64	67	63	65
J-MetS, *n* and %	51	53	43	44
ATPIII-MetS, *n* and %	43	45	35	36
Energy intake†, median and IQR				
Whole day (kcal)	2046	1897–2202	2067	1893–2187
Breakfast (kcal)	377	230–487	365	235–529
Lunch (kcal)	734	606–839	738	622–849
Dinner (kcal)	900	866–933	896	854–932
Carbohydrate intake, median and IQR				
Whole day (g)	270	247–292	275	250–302
Breakfast (g)	60	37–77	56	33–89
Lunch (g)	103	88–116	104	88–118
Dinner (g)	114	103–124	116	102–128
Protein intake, median and IQR				
Whole day (g)	75	72–78	74	71–77
Breakfast (g)	12	8–15	11	8–15
Lunch (g)	23	20–26	22	20–26
Dinner (g)	36	35–36	36	36–36
Fat intake, median and IQR				
Whole day (g)	61	56–68	62	54–67
Breakfast (g)	9	5–13	9	4–13
Lunch (g)	21	17–26	21	16–25
Dinner (g)	28	27–30	28	27–30
Fibre intake, median and IQR				
Whole day (g)	12	11–13	12	11–14
Breakfast (g)	2	2–3	2	1–3
Lunch (g)	4	3–5	4	3–5
Dinner (g)	6	6–7	6	6–7

MHLW-MetS, the Ministry of Health, Labor and Welfare definition of metabolic syndrome^(^ ^10^ ^)^; PSMetS, personal support lifestyle education program for the treatment of metabolic syndrome; IQR, interquartile range (25th percentile–75th percentile); MetS, metabolic syndrome; J-MetS, the Examination Committee of Criteria for ‘Metabolic Syndrome’ in Japan definition of metabolic syndrome^(^ ^9^ ^)^; ATPIII-MetS, the National Cholesterol Education Program Adult Treatment Panel III definition of metabolic syndrome^(^ ^8^ ^)^.

* Assessed as fasting plasma glucose ≥100 mg/dl or glycated Hb ≥5·6 %.

† 1 kcal=4·184 kJ.

The outcomes were measured at baseline (before randomisation) and at the end point (1 year after), and the changes from baseline at 1 year were the effect sizes of the present study.

### Study hypothesis

The hypothesis of the study was that participants with MHLW-MetS or high risk for MHLW-MetS in the PSMetS group would exhibit larger reductions in one or more of the MHLW-MetS risk factors for MHLW-MetS (R1 to R5), compared with the usual care group, 1 year after the randomisation.

### Sample size

The sample size required for the study was calculated assuming an effect size of about 0·47. Because we had little information about the changes to expect in the number of risk factors, we drew on our experience of conducting similar studies and calculated the effect size by estimating improvement rates of 20 % for the intervention group and 5 % for the usual care group. Using a two-sided significance level of 5 % and a power of 80 %, and assuming a dropout rate of 20 %, a sample size of 180 was estimated to be sufficient.

### Data management and data monitoring

We captured all study data in a Microsoft^®^ Excel file. Validation rules for each case record had been pre-specified and included range checks so that inaccuracies in data collection could be identified early. A query was raised for values outside the allowed range or if data were missing. An independent study monitor audited the records of each randomised participant and ensured that the study documentation was current, that record-keeping adhered to the study protocol and was in accordance with regulatory requirements, and that handling of the dietary lifestyle education programme was appropriate.

### Statistical analysis

Analyses were performed on an intention-to-treat (ITT) basis^(^ ^25^ ^)^ according to the treatment group allocated at randomisation. The full analysis set was included and the last observation carried forward method was used.

The effect size for continuous variables was calculated as the mean change from baseline and odds ratios were used for categorical variable. Sensitivity assessment was performed by per-protocol set (PPS) analysis with the complete data set.

The differences in the changes from baseline for the number of risk factors for MHLW-MetS (R1 to R5; the primary outcome) and the proportion of participants with MetS as assessed by the other definitions (the secondary outcomes) between the treatment and control groups were assessed by the Wilcoxon’s rank-sum test for the crude model and by van Elteren’s test^(^ ^26^ ^)^ for the adjusted models. ANCOVA for other continuous outcome measures and logistic models for categorical outcome measures were used to examine the effects of the intervention by adjusting for baseline values. Namely, a crude model, a baseline-adjusted model and a multivariate-adjusted model (adjusted for age, baseline value and baseline total number of risks for MHLW-MetS or proportion of MetS as assessed by several definitions) were employed. Although some variables with ordinal values were treated as continuous variables for the purposes of the ANCOVA, we examined the normality of data distributions visually and performed Bartlett’s test for the homogeneity of variances.

All tests for significance were conducted using a two-tailed approach with a 5 % significance level. All statistical analyses were performed using the statistical software package SAS version 9.4 for Windows. The trial and the report were designed with consideration of the relevant CONSORT (Consolidated Standards of Reporting Trials) group guidelines^(^ ^27^ ^)^.

## Results

### Trial flow and baseline characteristics

Overall, 406 workers with MHLW-MetS or a high risk for MHLW-MetS were recruited from nine companies in Tokyo and 193 eligible participants who had provided written informed consent were enrolled into the study between June 2010 and December 2013. Follow-up concluded in January 2015, 12 months after the last participant was randomised, as planned.

**Fig. 1:**
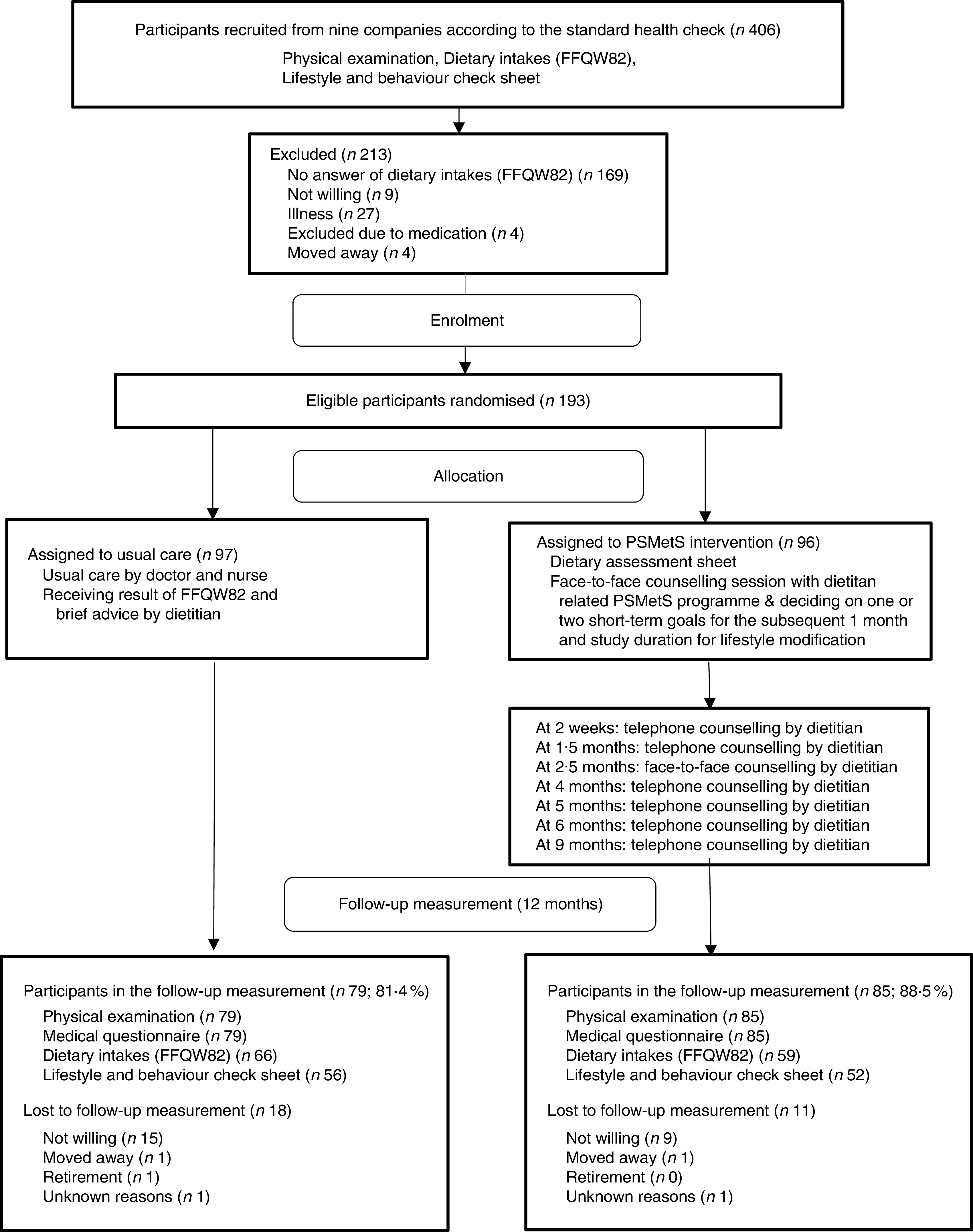
Study flow (PSMetS, personal support lifestyle education program for the treatment of metabolic syndrome)

Of the 193 randomised participants, 164 participants (85·0 %) completed the 1-year follow-up. The proportion of participants who were lost to follow-up was larger in the control group (18·6 %) compared with the intervention group (11·5 %), and the main reason was ‘not willing’ for both groups. The details are shown in Fig. 1. The participants were office workers (*n* 56), products sales workers (*n* 77), automatic line workers (*n* 25), shift workers (*n* 23) and marketing workers (*n* 12). All participants received a physical examination at an annual health check-up. They were asked to complete the FFQW82 and the lifestyle and behaviour check sheet before randomisation. Only 125 participants completed the FFQW82 at 1 year after check-up (Fig. 1).

Table 2 shows the baseline characteristics of the participants in the PSMetS group and usual care group. The PSMetS and usual care groups had similar characteristics at baseline. The overall mean age of the participants was 41·0 years. One hundred and twenty-seven (65·8 %) participants were included on the basis of diagnosis with MHLW-MetS, and sixty-seven (34·7 %) participants were included as having a high risk for MHLW-MetS. The mean number of risk factors for MetS at baseline was 4·3 (sd 0·9) for the PSMetS group and 4·1 (sd 0·9) for the usual care group.

### Primary outcome

Table 3 shows the changes in the number of risk factors for MHLW-MetS from baseline to 1 year in the participants of the PSMetS and usual care groups. The crude, baseline-adjusted and multivariate-adjusted mean differences in the number of risk factors for MHLW-MetS in the PSMetS group were not significant compared with the number of risk factors in the usual care group by the Wilcoxon rank-sum or van Elteren test (*P*=0·051, 0·075 and 0·085, respectively) in the ITT analyses. On the other hand, PPS analyses showed a significant reduction in the number of risk factors for all participants included in the per-protocol set (*P*=0·029, 0·038 and 0·044, respectively).

**Table 3:** Difference between changes from baseline to 1 year in the PSMetS and control groups: MetS and its components (ITT and PPS); male workers from nine companies in metropolitan Tokyo, Japan, enrolled June 2010–December 2013

	ITT analysis (*n* 193)									PPS analysis (*n* 164)								
	Crude			Baseline-adjusted			Multivariate-adjusted*			Crude			Baseline-adjusted			Multivariate-adjusted*		
Variable	Mean	95 % CI	*P* value	Mean	95 % CI	*P* value	Mean	95 % CI	*P* value	Mean	95 % CI	*P* value	Mean	95 % CI	*P* value	Mean	95 % CI	*P* value
No. of risk factors for MHLW-MetS			0·051§			0·075║			0·085║			0·029§			0·038║			0·044║
Proportion of MetS																		
MHLW-MetS			0·051§			0·031║			0·035║			0·036§			0·025║			0·024║
J-MetS			0·047§			0·085║			0·091║			0·031§			0·066║			0·063║
ATPIII-MetS			0·017§			0·046║			0·056║			0·008§			0·046║			0·053║
Components of MetS																		
Waist circumference (cm)	−2·2	−3·4, −1·0	<0·001	−2·1	−3·3, −0·9	0·001	−2·1	−3·2, −0·9	0·001	−2·5	−3·8, −1·1	<0·001	−2·3	−3·6, −0·9	0·001	−2·3	−3·7, −0·9	0·001
BMI (kg/m^2^)	−0·6	−0·9, −0·2	0·002	−0·5	−0·9, −0·2	0·002	−0·5	−0·9, −0·2	0·005	−0·6	−1·0, −0·2	0·002	−0·6	−1·0, −0·2	0·003	−0·6	−1·0, −0·2	0·006
Log-transformed TAG (mg/dl)	−0·1	−0·2, 0·0	0·125	−0·1	−0·2, 0·1	0·336	0·0	−0·2, 0·1	0·399	−0·1	−0·2, 0·0	0·124	−0·1	−0·2, 0·1	0·336	0·0	−0·2, 0·1	0·431
HDL cholesterol (mg/dl)	−0·7	−2·1, 0·8	0·376	−0·5	−1·9, 0·9	0·498	−0·4	−1·8, 1·0	0·558	−0·7	−2·4, 1·0	0·420	−0·5	−2·1, 1·2	0·577	−0·5	−2·1, 1·1	0·549
Systolic blood pressure (mmHg)	−1·7	−5·0, 1·6	0·311	−1·4	−4·6, 1·8	0·397	−1·4	−4·7, 1·8	0·389	−1·8	−5·7, 2·1	0·371	−1·3	−5·1, 2·4	0·485	−1·4	−5·2, 2·5	0·478
Diastolic blood pressure (mmHg)	−0·9	−3·6, 1·9	0·525	−0·5	−3·1, 2·1	0·698	−0·4	−3·1, 2·2	0·758	−1·0	−4·2, 2·2	0·542	−0·6	−3·6, 2·5	0·707	−0·5	−3·6, 2·6	0·737
Glucose intolerance (mg/dl)† ‡	1·6	0·9, 2·7	0·131	1·9	0·9, 3·9	0·073	1·8	0·9, 3·7	0·120	1·8	0·9, 3·3	0·075	2·1	1·0, 4·5	0·044	2·0	0·9, 4·2	0·075
Smoking status‡	1·5	0·9, 2·8	0·150	1·0	0·00, 999	1·000	1·0	0·00, 999	1·000	1·5	0·8, 2·8	0·245	1·0	0·001, 999	1·000	1·0	0·00, 999	1·000

PSMetS, personal support lifestyle education program for the treatment of metabolic syndrome; MetS, metabolic syndrome; ITT, intention-to-treat; PPS, per-protocol set; MHLW-MetS, the Ministry of Health, Labor and Welfare definition of metabolic syndrome^(^ ^10^ ^)^; J-MetS, the Examination Committee of Criteria for ‘Metabolic Syndrome’ in Japan definition of metabolic syndrome^(^ ^9^ ^)^; ATPIII-MetS, the National Cholesterol Education Program Adult Treatment Panel III definition of metabolic syndrome^(^ ^8^ ^)^. PSMetS (*n* 96) *v*. control (*n* 97) for ITT; PSMetS (*n* 85) *v*. control (*n* 79) for PPS.

* ANCOVA, adjusted for baseline, ‘MHLW-MetS level’ and age.

† Assessed as fasting plasma glucose ≥100 mg/dl or glycated Hb ≥5·6 %.

‡ Odds ratio for resolution.

§ Wilcoxon rank-sum test.

║ van Elteren test, adjusted for baseline and ‘MHLW-MetS level’.

### Secondary outcomes

The intervention group had a significantly greater reduction from baseline to 1 year in the proportion of MHLW-MetS in both the ITT (baseline-adjusted: *P*=0·031; multivariate-adjusted: *P*=0·035) and PPS (baseline-adjusted: *P*=0·025; multivariate-adjusted: *P*=0·024) analyses. The proportions of MetS based on the J-MetS or ATPIII-MetS diagnostic criteria also tended towards a greater reduction in the intervention group, although some of those differences were not statistically significant for the baseline- and multivariate-adjusted measures. Among the components, the PSMetS group showed significantly greater reductions in mean waist circumference (baseline-adjusted: −2·1 cm, 95 % CI −3·2, −0·9 cm, *P*=0·001) and BMI (baseline-adjusted: −0·5 kg/m^2^, 95 % CI −0·9, −0·2 kg/m^2^, *P*=0·002) by ITT analysis. Highly similar results for waist circumference and BMI were obtained in the analyses using the other models and PPS. No significant effects were observed on serum lipids, blood pressure and glucose.

Table 4 shows the change from baseline to 1 year in energy and nutrient intakes in the PSMetS group (*n* 59) and the usual care group (*n* 66) as determined by PPS analysis. Significantly greater mean reductions from baseline to 1 year in the PSMetS group compared with the usual care group were found for energy intake during dinner (the evening meal; −0·03 kcal, 95 % CI −0·06, −0·00 kcal, *P*=0·025) and fat intake during the whole day (−0·07 g, 95 % CI −0·12, −0·01 g, *P*=0·016) and at dinner (−0·04 g, 95 % CI −0·07, −0·01 g, *P* <0·001) in the crude analyses. However, the corresponding values from the baseline-adjusted and multivariate-adjusted analyses were not significant. On the other hand, protein intake during dinner was shown to be significantly increased in the PSMetS group according to crude (0·01 g, 95 % CI 0·00, 0·02 g, *P*=0·020) and multivariate-adjusted (0·01g, 95 % CI 0·00, 0·01 g, *P*=0·048) measures.

**Table 4:** Difference between changes from baseline to 1 year in the PSMetS and control groups: energy and nutrient intakes (PPS analysis, *n* 125); male workers from nine companies in metropolitan Tokyo, Japan, enrolled June 2010–December 2013

	Crude			Baseline-adjusted			Multivariate-adjusted		
Variable	Mean	95 % CI	*P* value	Mean	95 % CI	*P* value	Mean	95 % CI	*P* value
Energy*									
Whole day (kcal)	−0·04	−0·08, 0·01	0·094	−0·02	−0·06, 0·02	0·229	−0·02	−0·06, 0·02	0·238
Breakfast (kcal)	0·32	−0·09, 0·73	0·130	0·26	−0·14, 0·66	0·206	0·26	−0·14, 0·67	0·203
Lunch (kcal)	0·07	−0·15, 0·29	0·539	0·07	−0·15, 0·29	0·547	0·07	−0·15, 0·28	0·537
Dinner (kcal)	−0·03	−0·06, −0·00	0·025	−0·02	−0·04, 0·00	0·100	−0·02	−0·04, 0·00	0·104
Carbohydrate									
Whole day (g)	−0·03	−0·08, 0·03	0·290	−0·03	−0·08, 0·02	0·211	−0·04	−0·09, 0·01	0·140
Breakfast (g)	0·20	−0·10, 0·50	0·186	0·18	−0·11, 0·48	0·220	0·14	−0·15, 0·44	0·339
Lunch (g)	0·04	−0·12, 0·20	0·608	0·05	−0·11, 0·21	0·559	0·03	−0·13, 0·20	0·690
Dinner (g)	−0·05	−0·12, 0·02	0·138	−0·05	−0·12, 0·01	0·093	−0·06	−0·12, 0·01	0·080
Protein									
Whole day (g)	−0·02	−0·04, 0·01	0·156	−0·01	−0·04, 0·01	0·245	−0·02	−0·04, 0·01	0·186
Breakfast (g)	0·09	−0·08, 0·27	0·300	0·10	−0·08, 0·27	0·270	0·08	−0·09, 0·26	0·358
Lunch (g)	0·06	−0·06, 0·18	0·356	0·06	−0·06, 0·18	0·307	0·05	−0·07, 0·17	0·400
Dinner (g)	0·01	0·00, 0·02	0·020	0·01	0·00, 0·01	0·056	0·01	0·00, 0·01	0·048
Fat									
Whole day (g)	−0·07	−0·12, −0·01	0·016	0·11	−0·07, 0·30	0·233	−0·05	−0·10, 0·00	0·051
Breakfast (g)	0·09	−0·11, 0·29	0·372	0·03	−0·08, 0·15	0·548	0·10	−0·09, 0·29	0·295
Lunch (g)	−0·01	−0·19, 0·16	0·895	0·00	−0·16, 0·16	0·991	0·00	−0·17, 0·16	0·978
Dinner (g)	−0·04	−0·07, −0·01	<0·001	−0·02	−0·05, 0·00	0·068	−0·02	−0·05, 0·00	0·066
Fibre									
Whole day (g)	0·01	−0·05, 0·07	0·758	0·01	−0·05, 0·07	0·676	0·01	−0·05, 0·07	0·828
Breakfast (g)	0·03	−0·09, 0·15	0·640	0·03	−0·08, 0·15	0·548	0·02	−0·09, 0·14	0·691
Lunch (g)	0·07	−0·04, 0·17	0·215	0·06	−0·04, 0·16	0·267	0·05	−0·06, 0·15	0·366
Dinner (g)	0·00	−0·03, 0·03	0·935	0·00	−0·03, 0·03	0·977	0·00	−0·03, 0·03	0·920

PSMetS, personal support lifestyle education program for the treatment of metabolic syndrome; PPS, per-protocol set. PSMetS (*n* 59) *v.* usual care (*n* 66) for PPS analyses. ANCOVA, adjusted for baseline, type and age.

* 1 kcal=4·184 kJ.

Table 5 shows the changes from baseline to 1 year in the PSMetS group (*n* 52) and the usual care group (*n* 56) for the items of the lifestyle and behaviour check sheet. The PSMetS group showed significantly greater increases in the number of participants having a high level of health consciousness (mean=0·33, 95 % CI 0·07, 0·58, *P*=0·012), in the frequency of reducing energy intake at dinner (mean=1·15, 95 % CI 0·61, 1·68, *P*<0·001), in the frequency of two portions of vegetables intake at each meal (mean=0·77, 95 % CI 0·27, 1·27, *P*=0·003) and in the frequency of physical activity undertaken per week (mean=0·60, 95 % CI 0·20, 0·99, *P*=0·003) after adjusting for baseline by ANCOVA. The results of Bartlett’s test were significant for the frequency of two portions of vegetables intake at each meal (*P*=0·031) and the frequency of eating staple foods (*P*=0·039).

**Table 5:** Difference between changes from baseline to 1 year in the PSMetS and control groups: items of the lifestyle and behaviour check sheet (PPS, *n* 108); male workers from nine companies in metropolitan Tokyo, Japan, enrolled June 2010–December 2013

	Baseline values												
	PSMetS			Control			Crude			Baseline-adjusted*			Bartlett’s test
Variable (higher value denotes better)	*n*	Mean	sd	*n*	Mean	sd	Mean	95 % CI	*P* value	Mean	95 % CI	*P* value	*P* value
Health consciousness	52	2·6	0·9	57	2·8	0·8	0·23	−0·04, 0·50	0·093	0·33	−0·07, 0·58	0·012	0·130
Frequency of reducing energy intake at dinner	52	3·3	1·6	56	2·8	1·4	0·65	−0·02, 1·32	0·056	1·15	0·61, 1·68	<0·001	0·836
Frequency of two portions of vegetables intake at each meal	52	2·8	1·4	56	2·8	1·6	0·79	0·2, 1·4	0·010	0·77	0·27, 1·27	0·003	0·031
Frequency of eating staple foods	52	4·7	1·6	56	4·4	1·9	0·25	−0·44, 0·95	0·471	0·49	−0·07, 1·05	0·085	0·039
Frequency of physical activity undertaken per week	52	2·5	1·0	56	2·4	1·1	0·54	0·1, 1·0	0·014	0·60	0·20, 0·99	0·003	0·932

PSMetS, personal support lifestyle education program for the treatment of metabolic syndrome; PPS, per-protocol set.

* ANCOVA.

## Discussion

### Principal findings

Our findings indicated that the PSMetS group showed significant reductions in the number of risks for MHLW-MetS for PPS, although it was not significant for ITT, a greater frequency of resolution from MHLW-MetS for ITT and PPS, and greater reductions in waist circumference and BMI for ITT and PPS (Table 3). The PSMetS group also showed improvements in lifestyle factors, including reduced energy and fat intakes during the whole day and during dinner (Table 4), a reduced energy intake at dinner, an increased frequency of eating two portions of vegetables during each meal, and an increased frequency of physical activity per week (Table 5). The result of Bartlett’s test was significant for the reduction of energy intake at dinner. However, considering that the standard deviation was not drastically large and that ANCOVA has a certain degree of robustness for the analysis of non-normally distributed data, the use of ANCOVA might still be acceptable. We interpreted these results to indicate that counselling by a registered dietitian (i.e. how to select optimal foods and portion sizes, limit salt intake, consume fibre-rich foods, perform physical activity and behaviour modifications) affected the participants’ behaviour and reduced their number of risk factors for MetS. The results of the study thus indicated that the PSMetS intervention appeared to have positive effects for participants. Behavioural intervention for MetS may also be implemented by other groups performing counselling and health assessments for MetS in Japan. Lifestyle modification is the cornerstone of treatment for people with MetS.

### Comparison with other studies

Our findings showed that the PSMetS intervention led to a reduction in the mean number of risk factors for MHLW-MetS. Many studies to date have focused on treating certain individual component(s) of MetS, such as obesity, while our study focused on treating MetS as a whole.

Previous studies investigating the effectiveness of lifestyle modification approaches for individuals with MetS can be divided into two types: those using dietary intervention only and those using combined dietary and exercise intervention^(^ ^11^ ^)^. Several studies have been published that correspond to the latter^(^ ^15^ ^–^ ^18^ ^)^ and former^(^ ^12^ ^–^ ^14^ ^,^ ^28^ ^)^ categories. Of these studies, representative changes from baseline to the end of study in the mean number of risk factors for MetS were as follows: −0·4 at 6 months^(^ ^18^ ^)^ and −0·4 at 10 months^(^ ^17^ ^)^, which were greater reductions than that found in our study of −0·6 at 12 months. Some of these studies reported changes in waist circumference (cm) of −4·0 at 6 months^(^ ^12^ ^)^ and −4·6 at 12 months^(^ ^15^ ^)^, which were broadly similar to our finding of −2·3 at 12 months.

Lifestyle modification intervention based on assessment mainly using the FFQW82, the lifestyle and behaviour check sheet, and the dietary assessment sheet had impacts on the behaviour of excessive eating at night among male workers with MetS. The available evidence indicates that excessive eating at night can increase insulin resistance^(^ ^29^ ^)^. The PSMetS programme aimed to improve central obesity by reducing energy and fat intakes, promoting optimum protein intake during dinner, improving post-meal plasma glucose by increasing the amount of vegetable intake per meal, and increasing the amount of exercise undertaken by the participants. Comparison with other studies showed that lifestyle modifications for reducing MetS risk factors were successfully achieved in line with our initial study aims.

On the other hand, significant effects of the intervention on serum lipids, blood pressure and glucose were not observed in the present study. In the PSMetS programme, reduction of weight (i.e. reduction of BMI and waist circumference) was the first aim. Weight reduction is related to the improvement of central obesity and might also be expected to improve other risk factors. Therefore, it may ultimately reduce the number of risk factors of MetS. Although the changes were not significant, blood pressure and serum lipid measurements improved somewhat during the intervention period. As in a similar study^(^ ^30^ ^)^, only BMI showed a significant reduction in the intervention group after 1 year, while at the end of the study intervention (average duration 4·2 (sd 2·0) months), the prevalence of MetS was significantly lower in the intervention group. In order to observe significant effects on blood pressure and serum lipids, an intensive care intervention period of longer than the 6 months performed in the present study might be needed. However, we could consider another possible reason for the lack of an observed significant change in blood pressure and serum lipids, which is that these measures were good at baseline. Further study is warranted to determine whether such effects can be observed over a longer period.

### Strengths and limitations of the study

Our randomised trial had several strengths. One of the strengths of the present study is that it was the first RCT in Japan to have evaluated the use of a lifestyle education counselling programme in combination with specific dietary assessments in male workers. The counselling was focused on reducing participants’ risk factors for MetS. Therefore, registered dietitians could focus on recommending dietary improvements for participants based on an understanding of optimum dietary intakes garnered from assessments of energy and vegetable intakes during each meal, with particular emphasis on combating overeating of fats, rice, sweets, and so on. Furthermore, to establish evidence-based nutrition education, examining the effects of the PSMetS programme presented by registered dietitians in a counselling-based study is important. The study was designed to examine the number of risk factors of MHLW-MetS together with individual MHLW-MetS risk factors. Central obesity, the most prevalent manifestation of MetS, is a sign of dysfunctional adipose tissue and is of central importance in clinical diagnosis^(^ ^31^ ^)^. Reduced adiponectin levels can be caused by interactions between genetic factors, such as SNP276 in the adiponectin gene, and environmental factors, such as a high-fat diet, too much energy intake late at night and a sedentary lifestyle^(^ ^32^ ^)^. Especially, a meta-analysis of nine RCT showed the effects of fat intake on BMI and waist circumference^(^ ^33^ ^)^. In addition, a systematic review and meta-analysis showed that, compared with an energy-restricted low-fat diet, an isoenergetic prescribed high-protein and low-fat diet provides a modest advantage for reducing body weight and fat mass^(^ ^34^ ^)^. These studies supported the positive effects of PSMetS for participants with MHLW-MetS or a high-risk for developing MHLW-MetS.

There are some limitations of the study design. First, the success of this programme was to some extent dependent on the skill of the dietitians. To address this issue, we developed a training process for the registered dietitians to undertake before the start of the randomised study. Furthermore, the design of the dietary assessment sheet, on which the items were ranked according to priority, was intended to help to standardise the advice given to participants by the dietitians. Regarding the intervention fidelity, the dietitians had to report on their delivery of the interventions. Although we cannot deny the possibility of diversity in delivery among the different dietitians, this may work as an assessment of the consistency of delivery of the intervention by the dietitians. We assessed the dietitians’ reports and concluded that while there was some variation among the dietitians, they all delivered the intervention within the guidelines provided during their training and on the dietary assessment sheet. The amount of variation we identified was no greater than expected. These observations indicated that the methodological approach we used, including the provision of training and instructions to the dietitians involved, could produce an acceptable level of consistency in the delivery of the PSMetS intervention. In addition, different dietitians saw the usual care and intervention participants and this could avoid a source of bias. Second, the study was not blinded because of the nature of the lifestyle intervention. Therefore, it was possible that workers receiving usual care and those receiving the intervention at the same company spoke to each other about the dietary treatments they were receiving. Although we cannot deny the possibility, it may not largely affect the results, although if there were an effect, it may be expected to reduce the difference observed between the groups. Third, we cannot deny the possibility of selection bias. To avoid this, we asked nurses to recruit all the participants in order. Fourth, the dropout rate was somewhat larger in the control group than in the intervention group. This may because the frequency of contact with the dietitian was lower and it caused less concern to participants to discontinue in the control group. This could be interpreted as an intervention effect. However, we should interpret the results carefully, since we cannot differentiate the quality, quantity and frequency of the intervention effects. Fifth, the range of occupations of the study participants was limited. Therefore, the generalisability of the results is limited to Japanese male workers working in similar conditions. Further study is warranted to determine the effects of PSMetS in other MetS populations.

### What is already known on this topic

MetS is the leading risk factor for type 2 diabetes and CVD. A meta-analysis of RCT showed that lifestyle modification was effective in resolving MetS and reducing the number of risk factors for MetS.

### What the present study adds

The PSMetS intervention included providing the participants with assessments of their actual dietary practices of energy and nutrient intakes at each meal as revealed by the FFQW82. This approach was intended to increase participants’ motivations to improve these practices and to help them to recognise a need for behavioural modification. The findings showed that PSMetS reduced the number of risk factors for MHLW-MetS in participants with MHLW-MetS or a high risk of MHLW-MetS, compared with usual care only.

## Conclusions and policy implications

To date, there has been a lack of evidence from RCT regarding the effects of providing dietary and lifestyle intervention for Japanese men who have MHLW-MetS or a high risk of MHLW-MetS. Our trial fills this gap in the literature. Our results indicated that a combined dietary and lifestyle intervention programme reduced the mean number of risk factors for MHLW-MetS and increased the frequency of participants achieving resolution from MHLW-MetS. The test population showed a moderately high adherence rate to the PSMetS programme, which is a positive indication of its feasibility in broader clinical practice. Our study provides useful information not only for Japanese MetS patients but also for patients in other countries. This manuscript will also help to improve decision making in medical practice, policy, education and future research, and will be important to international general medical readers.
